# Teaching the chemical elements in biochemistry: Elemental biology and metallomics

**DOI:** 10.1002/bmb.21614

**Published:** 2022-02-26

**Authors:** Wolfgang Maret, Philip Blower

**Affiliations:** ^1^ Department of Biochemistry, Faculty of Life Sciences and Medicine King's College London London UK; ^2^ Department of Nutritional Sciences, School of Life Course and Population Sciences, Faculty of Life Sciences and Medicine King's College London London UK; ^3^ School of Biomedical Engineering and Imaging Sciences, Faculty of Life Sciences and Medicine King's College London London UK

**Keywords:** bioelements, biometals, chemical elements

## Abstract

Biochemistry primarily focuses on the non‐metal chemical elements carbon, oxygen, nitrogen, hydrogen, sulfur, and phosphorus in the four groups of building blocks (sugars, lipids, amino acids, and nucleotides) and the corresponding macromolecules. However, at least 10 essential chemical elements of life are metals. This article discusses the consequences of such a bias, presents current knowledge that over 20 chemical elements are required for life, and makes a case for—and suggests benefits of—teaching *elemental biology* alongside molecular biology and biochemistry, and inorganic chemistry in addition to organic chemistry. A relatively new interdisciplinary field, metallomics, has the potential to be a platform for integration when added to glycomics, lipidomics, proteomics, and genomics. It would fill a major gap in contemporary education, be relevant for many areas of science, and facilitate the teaching of important principles of chemistry in the biological sciences, thus helping students to gain a broader understanding of life processes from the molecular to the systemic biology level.

## Graphical abstract

The presence of many essential chemical elements ‐ shown in a Periodic Table for humans ‐ in addition to many non‐essential elements requires teaching elemental biology.

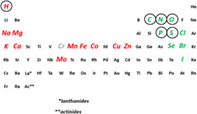

## 1 INTRODUCTION: A BIAS IN BIOCHEMISTRY TOWARDS THE BULK NON‐METAL ELEMENTS AND THE SIGNIFICANCE OF METAL ELEMENTS FOR LIFE

Physics, chemistry, and biology seem to be rather well‐defined disciplines for many of us. In contrast, the hybrid disciplines in the life sciences are a bit more difficult to grasp in their contents and coverage. A brief look at chronology should be instructive. With the synthesis of urea in 1828, Friedrich Wöhler dispelled the belief that only living organisms can synthesize organic compounds. The discovery refuted vitalism and pioneered organic chemistry, which later branched off into physiological chemistry and biological chemistry. In the first half of the 20th century, the chemistry of life processes became *biochemistry* with the discoveries of vitamins and minerals as coenzymes and cofactors, the functions and structures of enzymes and their involvement in transformations of metabolites in metabolic pathways and the assembly of the low‐molecular‐weight molecules of life into macromolecules. In the second half of the 20th century, *molecular biology* emerged as a discipline. The discovery of DNA/RNA functions provided the basis for: a genetic code for proteins, a molecular explanation for genetics, and the development of technologies of cloning and the engineering of genes. *Cell biology* also emerged as a discipline. It pursues an understanding of how signal transduction pathways and networks—and cellular compartments in eukarya—co‐operate in organizing the complexity, versatility, and robustness of cellular function. Overall, advances in techniques and methods allowed fields to develop from small molecules to larger ones and ever more complex structures, filling the gap between the nanoscale and the microscale. In present day terminology, it was a bottom‐up approach that slowly revealed the hierarchical organization in biological structures from isolated molecules to macromolecular complexes, cells, and organisms.

**FIGURE 1:**
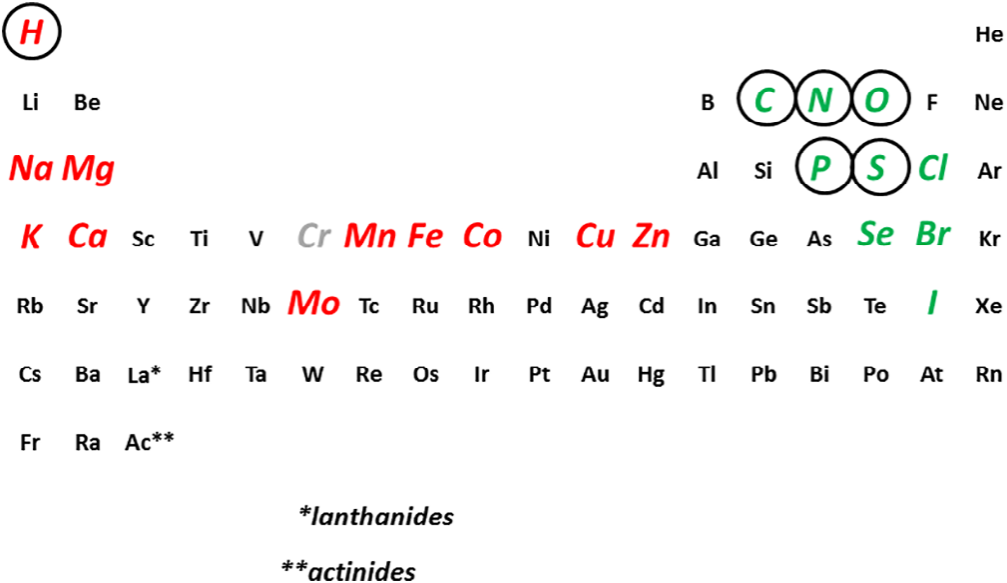
The Biological Periodic Table of the Elements. The essential elements are given for humans, including the uncertainties about the role of chromium in biochemistry (shown in gray). Metals (including hydrogen) are shown in red and non‐metals in green. SPONCH elements are circled. The acronym SPONCH is readily recognizable when reading backwards from sulfur (S)

Biochemistry as a core discipline in the life sciences and medicine teaches the structure, function, and metabolism of the four building blocks: sugars (carbohydrates), fats (lipids), amino acids, and nucleotides and how they combine to form the biological macromolecules, polysaccharides, membrane bilayers, proteins, and nucleic acids. It involves the elements carbon (C), hydrogen (H)—theoretically considered as a metal because it has the properties of one at very high pressures—nitrogen (N), oxygen (O), phosphorus (P), and sulfur (S), collectively referred to with the acronym SPONCH (Sulfur, Phosphorus, Oxygen, Nitrogen, Carbon, Hydrogen). Biochemistry combines the study of ideas and compounds from the fields of both organic and inorganic chemistry. When teaching biochemistry, drawing a sharp dichotomy of organic versus inorganic chemistry or omitting the study of or, at least, mention of appropriate sections of inorganic as well as organic chemistry is inappropriate and unhelpful. It does not do justice to the diversity of chemical elements used by and essential to living organisms.^1^ The dichotomy has survived though not necessarily passed the test of time. Its propagation in the separate fields of bioinorganic and bioorganic chemistry requires understanding of the implications. When biochemical studies focus exclusively on non‐metals, they focus only on a pocket on the right side of the Periodic Table of the Elements. However, at least 10 metals on the left side and in the center of the Periodic Table have crucial functions in human life processes. These include sodium, potassium, magnesium, calcium, manganese, iron, molybdenum, cobalt, zinc, and copper. Thus, in addition to SPONCH, the above six bulk non‐metal elements, 10 metal elements are essential, bringing the total count to 20 essential chemical elements in humans when including the additional four non‐metal elements: the halogens chlorine, bromine, iodine and the chalcogen selenium (Figure 1).

For other forms of life, yet other metal elements are essential: vanadium, tungsten, nickel, and even cadmium and some of the lanthanides. It is not clear whether this list of essential chemical elements is complete. The essential nature of some elements was discovered rather recently, for example, bromine for humans and lanthanides for some bacteria in 2014.^2^, ^3^ Furthermore, uncertainty persists about the indispensability and functions of some, for example, chromium, for which the structure of its biological complex and its precise molecular target(s) remain unknown.^4^

Without metal ions life would not be possible or would not even have evolved from interactions of the biosphere with the atmosphere, geosphere, and hydrosphere. Metal ions are critically important in the many metabolic processes involving gases. In human physiology, oxygen chemistry is linked to iron and copper, nitric oxide chemistry to iron, and carbon dioxide chemistry to zinc. In plants, oxygen evolution occurs by a manganese cluster cofactor catalyzing the oxidation of water. In some microorganisms, nitrogen fixation occurs via an iron/molybdenum cluster or, in some instances, an iron/vanadium cluster. Some microorganisms (methanotrophs) use or produce (methanogens) methane, using copper and nickel cofactors in the process.^5^ All this calls for a need for biochemistry and molecular biology courses to include some consideration of the biological roles of metals.

In textbooks of nutrition in particular, metals and the additional essential elements are often taught as minerals together with vitamins that cannot be synthesized. Clearly, we cannot synthesize any element and all essential elements must be supplied to an organism to grow and survive. Our diet, the air we breathe and the water we drink are the sources of the six bulk, non‐metal SPONCH elements in the same way they are for the other elements. The only difference is quantity not quality. The quantities of essential chemical elements present in a human body vary over at least seven orders of magnitude, varying from 43 kg oxygen down to 5 mg molybdenum.^6^ The low amounts of some elements are as essential as the high amounts of others. The concept of *trace elements* arose from the limited sensitivity of methods to analyze these elements in the past.^7^ Whether or not some are merely traces is not relevant for function or importance. Focusing on only the bulk elements in biochemistry, which make up for 97.5% of the composition of the human body, is pure chemical discrimination against an essential minority!

Between the metals and non‐metals in the Periodic Table are the metalloids, which is a poorly defined term for elements that have both metal and non‐metal characteristics. These include the following elements, which have either known or potential significance in biological systems: boron, silicon, germanium, arsenic, antimony, and tellurium. Boron is essential for plants and silicon is a constituent of the cytoskeleton of some marine organisms.^8^, ^9^ Metalloids do not seem to be essential for humans. Yet, some of them are present in living cells and how their presence affects biological processes needs to be examined.^10^

## 2 THE CONCEPT OF BIOMETALS/BIOELEMENTS INCLUDES BOTH ESSENTIAL AND NON‐ESSENTIAL ELEMENTS

Many additional metal elements are present in our body. Some, like strontium and rubidium, occur at higher concentrations than some essential elements. With modern instrumentation almost all the natural elements can be measured in biological tissue.^11^ Thus, all these elements are *bioelements* or *biometals* when present in biological organisms. Many of the non‐essential elements are considered non‐functional because we do not know what their functions are. It is a typical case of taking absence of evidence for evidence of absence. We therefore posit that the life scientist should have at least a rudimentary understanding of the properties of each chemical element, including why an element has been selected by evolution for its biological functions or has been rejected, what the ranges of concentrations are for the elements present, and which concentrations are considered adequate, safe, and tolerable before adverse effects occur. The understanding should include that essential bioelements can have adverse effects at excessively high concentrations, while low concentrations may lead to deficiencies. It should also be understood that non‐essential bioelements do not have such a bi‐phasic behavior. They have only adverse effects above a certain threshold. Paracelsus taught us that the dose determines whether an agent is a medicine or a poison. Therefore, before elements become toxic at higher concentrations, non‐essential elements can have beneficial functions, for example, fluoride for tooth health, and essential elements can have pharmacological functions. The fact that metabolic transformations occur is not an indication that an element is needed for an essential function, however. Non‐essential metals and metalloids can undergo specific metabolic transformations, for example, methylations for mercury or formation of arsenic compounds such as arsenosugars or arsenolipids.^12^, ^13^ There can also be a “per chance” incorporation of an essential elements into biological macromolecules. For example, facultative selenium incorporation into proteins can occur at higher dietary intake with functional consequences yet to be fully understood.^14^

Teaching what each chemical element can accomplish in a biological system, that is teaching *elemental biology*, differs throughout the tree of life. It is also noteworthy that, because of the limitations on pH values and redox potentials in living cells, not the entire possible chemistry of a chemical element is realized when it becomes a bioelement. A bioelement adopts only some of the chemical characteristics of an element to serve its biological functions. For example, zinc is a redox‐active element in chemistry, but in biology it always remains Zn(II) and its redox properties are not relevant.^15^ Or, as the case of chromium illustrates, different redox states have vastly different properties. Cr(III) is thought to be essential whereas Cr(VI) is toxic and carcinogenic.^16^

## 3 THE SIGNIFICANCE OF NON‐BULK ELEMENTS: INTEGRATING ELEMENTAL BIOLOGY INTO MOLECULAR BIOLOGY AND BIOCHEMISTRY

Given the significance of a rather large number of chemical elements in biology, education should aim at integrating the elemental biology of metals and additional appropriate non‐metals into molecular biology and biochemistry, and this is where important and instructive teaching is being called for. Each element has its individual chemistry and biochemistry, which not only concerns its function but also the specific ways that have evolved for acquisition, distribution, excretion, and systemic and cellular control. As is the case for biomolecules, one can formulate a metabolic pathway for each bioelement. Some metal ions are handled by prosthetic groups whose organic moieties must be synthesized, for example, protoporphyrin IX for iron, pyranopterin for molybdenum or corrin for cobalt, as is the case for vitamin B_12._ ^17^ In addition, microorganisms synthesize compounds with the specific function of acquiring metal ions from their environment, for example, the metallophores: siderophores for iron, zincophores for zinc, and chalcophores for copper.^18^, ^19^, ^20^ The metabolic pathways for the synthesis of such biological chelating agents are part of metal metabolism, too. And then there are specific transporter, storage, and sensor proteins as part of the system of homeostatic control for each metal ion. These proteins help ensure that each metal ion functions without interference from the others. They are separate from the proteins in which each metal ion serves Its primary functions. For zinc alone, at least three dozen proteins are necessary for import, export, cellular distribution, and control, each with its own remarkable biological chemistry for handling zinc(II) ions.^21^ Synthesizing all the ancillary proteins and factors that control metal metabolism and acquire metal ions from environments of sometimes extremely low metal abundance comes at significant energetic and entropic costs. As an example of the scope of metalloproteins, the catalytic power of at least half of all enzymes depends on metal ions. The enzyme with the highest turnover number—almost a million per second—is a zinc metalloenzyme, carbonic anhydrase. At least 3000 human proteins rely on zinc, more than every 10th protein.^22^ Zinc is required for *catalytic*, *structural*, or *regulatory* functions and its ubiquitous use has led to coining the expression that zinc galvanizes biology, epitomizing the notion that relatively small quantities of zinc (2–3 g) in a human body have a major impact on virtually all cellular functions.^21^, ^23^ *Catalytic* functions of zinc are observed in each class of enzymes with a preponderance of zinc as a cofactor of hydrolytic enzymes. *Structural* functions of zinc are very important for organizing protein domains. Zinc binding is part of building tertiary structure that is not possible to achieve otherwise, including linking cysteine side chains and thus substituting for disulfide bridges that are rare inside cells. It endows proteins with specific shapes and surfaces for interaction with other proteins, DNA/RNA, or lipids.^21^ In addition to their contribution to tertiary structure of proteins, zinc(II) ions can also serve as bridges between subunits of the same protein or different proteins, thus building quaternary and quinary structure in protein–protein interactions. At least 1000 transcription factors require zinc domains (called “zinc fingers”) when controlling gene expression. *Regulatory* functions of zinc involve transient binding to proteins. Thus, not only do a significant number of proteins control metal ions but metal ions also control proteins. And this entire process is fully integrated into the other metabolic and signaling pathways that govern the functioning of a cell. It means that many pathways that have no obvious relationship to a metal ion nevertheless are involved in the control of metal ions or underlie some control by metal ions.

Most established metal ion interactions concern proteins. As such, the field of biometals is protein‐centric, linking it closely to proteomics. Metalloproteins are a fundamental aspect of protein science. However, there is also interest in the interaction of biometals with other biomolecules and in the pool of biometal ions when they are not bound to proteins or in transit into, out of or within the cell. Characterization of the coordination environment of biometals in this non‐protein‐bound pool, however, is not straightforward due to the low concentrations of the ions involved and the multiplicity and mostly fast exchange kinetics of the biological ligands involved. Metal ions released from proteins or vesicular storage sites can be employed for intracellular regulation as second or third messengers. Ca^2+^ is well established as a signaling ion.^24^ More recently, sufficient evidence has accumulated to consider zinc(II) ions as signaling ions. They serve as intracellular messengers and extracellular messengers when secreted from cells by vesicular exocytosis.^25^ This fascinating aspect of biological regulation with metal ions involves about 12–15 orders of magnitude in the range of concentrations of different metal ions, from millimolar concentrations of sodium (Na^+^) and potassium (K^+^) controlling nerve impulses to micromolar/nanomolar concentrations of calcium (Ca^2+^) to nanomolar/picomolar concentrations of zinc (Zn^2+^) to even femtomolar/attomolar concentrations of copper ions, which are thought to be signaling ions, too.^26^ Transients of these metal ions control specific signaling events and physiological processes.

A final important point about the significance of metal ions is their role in biomineralization. A chapter in a textbook serves well as reference for the following examples.^27^ The role of calcium in the formation of bones and teeth is perhaps the most prominent example, the main constituent being calcium phosphate, hydroxyapatite [Ca_10_(PO_4_)_6_(OH)_2_]. Calcium carbonate is used in the formation of seashells in mollusks. Iron oxide is used as a magnetic sensor in the magnetosomes of bacteria. There are also gravity sensors that can include barium in addition to calcium. Marine organisms use silicates, and sometimes strontium sulfate in biomineralization. Ferritin, the iron storage protein, has a mineral core of iron(III) oxide (Fe_2_O_3_). While this is an example of a specific protein involved in mineralization of metal ions, the reverse is true, too: metal ions participate in the biocrystallization of proteins. Insulin is stored in the pancreatic β‐cells in a crystalline form as a hexamer with bound zinc and calcium ions.^28^ Biomineralization is also part of the interface between biochemical and geochemical cycles of metal ions. Bacteria have roles in in biomineralization, precipitating zinc sulfide (ZnS) nanoclusters by reducing sulfate to sulfide, and also dissolving the metal ion from a mineral such as ZnS by oxidizing the sulfur in an iron‐dependent reaction.^29^, ^30^

Last but not least, an interesting example of the importance of non‐metal bioelements is the biochemistry of selenium (a congener of sulfur in the Periodic Table) and of selenocysteine, sometimes referred to as the 21st amino acid. This amino acid is incorporated into at least 25 human selenoproteins (many of them oxidation–reduction enzymes) by the mRNA codon UGA, normally a stop codon.^31^ Other compounds important to selenium metabolism include: 1. the anion hydroselenide (HSe^−^) ‐ important in thyroid metabolism ‐ and 2. selenosugars and methylated species that are important for selenium secretion.

## 4 ADVANCES IN ANALYTICAL INSTRUMENTATION IMPORTANT TO STUDYING BIOMETALS: FROM METALS TO METALLOMES AND METALLOMICS

Advances in instrumental analytics, mainly inductively coupled plasma mass spectrometry, brought a new era to metal analytics. Mass spectrometric methods displaced atomic absorption and emission spectroscopies, which previously had been the workhorses in elemental analysis. They have improved sensitivity and suffer less interferences from the biological matrix. They are also suitable for imaging when mass spectrometry is coupled to ablation of material with lasers. Such imaging has provided evidence for uneven distribution of metal ions in some tissues and cells. These advances led to the definition of a new field, metallomics^32^ based on the concept of the metallome,^33^ the “ensemble of all the biomolecules in a system which bind a given metal ion”.^34^ Metallomics is a systems level approach to biometals and bioelements in the life sciences and adds a fifth pillar to the current four pillars of molecular sciences: glycomics, lipidomics, proteomics, and genomics.

## 5 INSIGHTS INTO USING METALLOMICS TO EXPAND THE BREADTH OF BIOLOGY AND CHEMISTRY COURSES AND TO HELP AND ILLUSTRATE CHEMICAL PRINCIPLES

Metallomics, as integrated into biological sciences, can introduce, enrich, and broaden interdisciplinary and multidisciplinary approaches to teaching. Uncharted areas in research and deficiencies in teaching elemental biology have led to large gaps in public discussions of such topics as the toxicity of aluminum in cooking ware, the effect of mercury in vaccines, or the efficacy of zinc supplements in treating Covid‐19 patients.^35^, ^36^, ^37^ Teaching chemical, physical, and biological principles and concepts as they apply to theory and practice can help understanding the foundations of the life sciences.

### 5.1 Chemistry for the biologist

Most of the organic chemistry of the bulk elements in biochemistry concerns covalent bonds. However, bulk non‐metal elements (oxygen, nitrogen, and sulfur) are the donor ligands of metal ions and utilize primarily ionic, inherently weaker interactions, although the outer electrons of sulfur, an element in period 3 of the Periodic Table, can access 3d orbitals and so sulfur can form strongly polarisable, somewhat covalent metal–ligand bonds. This highlights the need for teaching bonding affinities in both chemistry and biochemistry courses and suggests some interesting biologically important compounds to use as examples when discussing the need for a quantitative approach when applying chemical principles to explain biological processes. It is important for students to realize that understanding the reactivity of metal ions is crucial to understanding catalysis, redox reactions and free radical functions in biological processes. Coordination and physical chemistry (both thermodynamics and kinetics), ligand affinities (both association and disassociation rates), electronic properties (as applied to color) and spin states (as applied to magnetic sensitivities) enhance a student's understanding of structure and function in biochemistry.

### 5.2 Biology for the chemist

For the students of chemistry, it is important to understand the chemical principles as encountered in biological systems. Many publications in bioinorganic chemistry have limited relevance to biology as standard conditions (e.g., pH, temperature, and concentrations of reactants) are rarely found in living cells. A major focus in bioinorganic chemistry has been the coordination chemistry of biomolecules. The dynamic aspects of metal exchange and metal re‐distribution in living cells provide yet largely unexplored aspects of coordination chemistry.^38^

### 5.3 Other disciplines

Consideration and knowledge of elemental biology is important for applied biosciences such as harnessing biological processes for bioenergy, the bioindustry for waste conversion or food production and processing, synthetic biology and bioengineering, including biomimetic or bio‐inspired advanced functional materials.^39^

Metal ions also are employed in therapeutics and diagnostics.^40^, ^41^ For example, the radionuclides used in radioactive drugs (radiopharmaceuticals) to treat or image disease in nuclear medicine, are most often metals; their metallic identity may be unrelated to the pathology and relevant only to the detection process or may be directly relevant to the metal biology underlying the pathology being diagnosed.^42^, ^43^, ^44^

Metals have a wide range of actions, and their actions are taught in different ways: in nutrition as micronutrients, in pharmacology and medicinal chemistry as metallodrugs, and in toxicology as toxicants.

The metallomics of both ecology (as a field within biology) and environmental systems, metal speciation and chemical transformations of metals in the air, soil, and water is also of importance. We are living in a world that is increasingly challenged by pollution and climate change. It is therefore critically important to introduce the ecology of chemical elements in our curricula as it is at the center of the evolution of life and sustaining it in the future.

### 5.4 Practical and computational skills

Elemental biology needs a “toolbox” that is different from that in molecular biology. It generates additional opportunities for teaching and learning through project‐based and inquiry‐based learning in laboratory courses. In the field of bioinformatics, methods have been developed for mining (sic) genomes and proteomes for signatures of metal coordination environments in proteins, leading to a better understanding of structure/function relationships in metalloproteins. Predicting metal sites rather than isolating a protein and analyzing its metal content has developed into a critical metallomics tool to estimate the number of metalloproteins in an organism.^45^ How these techniques enable life scientists to characterize metal‐containing biomolecules helps students appreciate the power and utility of methodologies taught in related disciplines.
